# Diagnosis and Management of Stroke in Adults with Primary Brain Tumor

**DOI:** 10.1007/s11912-022-01280-6

**Published:** 2022-05-11

**Authors:** Edina Komlodi-Pasztor, Mark R. Gilbert, Terri S. Armstrong

**Affiliations:** National Institutes of Health/ National Cancer Institute/Neuro-Oncology Branch, 9030 Old Georgetown Road, Building 82, MD 20892 Bethesda, USA

**Keywords:** Ischemic stroke, Hemorrhagic stroke, Primary brain neoplasm, Low-grade tumor, High-grade cancer

## Abstract

**Purpose of Review:**

This article reviews the risk factors, clinical presentations, differential diagnosis, and the types of strokes frequently seen in patients with primary brain neoplasms. This includes a discussion of approaches with a review of the available literature and provides recommendations for primary and secondary prevention specific to this patient population.

**Recent Findings:**

Strokes in patients with brain tumors are often multifactorial. However, tailored approaches to stroke care are necessary to achieve optimal patient outcomes, AHA/ASA stroke guidelines provide little information on the management of stroke in cancer patients. A comprehensive algorithm for diagnosis for stroke in primary CNS tumor patients is proposed.

**Summary:**

Understanding the potential complex etiology of stroke in patients with brain tumors is essential to provide appropriate treatment and initiate optimal prevention measures early in the cancer treatment program. Optimal care therefore requires a comprehensive approach including a variety of specialists and healthcare providers.

## Introduction

Stroke is a neurologic emergency that requires rapid diagnosis and treatment as well as appropriate preventative measures to minimize risk of future events and to achieve maximal functional recovery along with improved quality of life. This is especially important for patients with primary brain tumors who have one of the highest relative risks for fatal stroke among all cancer patients and when compared to the general population [1]. Despite the recognized potentially poor outcome, leading organizations (including American Heart Association, American Stroke Association, and National Comprehensive Cancer Network) provide only limited guidance on the management of stroke in cancer patients. Furthermore, providers face additional challenges because stroke in cancer patients often evolve on complex underlying biology. In addition to the classic cerebrovascular risk factors (for example, hypertension, diabetes, hyperlipidemia, smoking, and atrial fibrillation), there are some unique processes, related to the diagnosis and complications of therapy that can be identified in primary brain tumor patients. As a result, prevention of stroke in brain cancer patients requires a multidisciplinary approach that should be started at the time of brain tumor diagnosis and continued throughout the patient’s lifetime.

In this article, we review the most commonly occurring risk factors that lead to either ischemic or hemorrhagic strokes in patients with primary brain tumors. We discuss the presentation and differential diagnoses of stroke in this patient population. We briefly review the basics of imaging diagnosis as well as the literature data on the acute treatment of stroke in brain tumor patients. Lastly, we discuss primary and secondary prevention strategies.

### Risk Factors

While there are few published manuscripts about stroke in primary brain tumor patients, the majority focus on identification of risk factors. Table 1 provides an overview of risk factors associated with comorbidities as well as the underlying primary brain tumor diagnosis and its treatment identified in this literature.

**Table 1:** Stroke risk factors in patients with primary brain neoplasm

Patients’ comorbidities	Hypertension
	Diabetes mellitus
	Hyperlipidemia
	Obesity/abdominal fat
	Smoking history
	Lack of physical activity
	Cardiovascular diseases
	Genetic risk factors
Tumor-related factors	Local vessel compression
	Vessel erosion by cancer
	Autocrine factors leading to coagulability
Surgery-related factors	Vessel injury during brain operation
	Anesthesia related cardiovascular changes
Radiation treatment–related factors	Microvascular changes in small vessels
	Microvascular changes in large vessels
	Aneurysm formation
Chemotherapy-related factors	Temozolomide
	Cisplatin
Related to additional treatment	Bevacizumab-related coagulopathy
	Corticosteroid
	Antithrombotic agents

It is well known that many of the common stroke risk factors seen in the general population directly contribute to vascular damage. Pre-existing vascular injuries make patients more vulnerable for stroke during and after brain tumor treatment. Hypertension, the most important risk factor for stroke, directly damages arteries and leads to microvascular and macrovascular changes [2]. Diabetes mellitus, especially uncontrolled disease, has been associated with macrovascular changes but it is also an independent risk factor for lacunar stroke by causing intracranial atherosclerosis, most commonly in the posterior circulation [3]. Hyperlipidemia promotes cervical artery or coronary atherosclerosis, predisposing the patient to atherothrombotic and cardioembolic stroke [4]. Lifestyle-related risk factors that can be improved by behavioral modifications include smoking, lack of physical activity, and obesity (most importantly abdominal fat) [5]. Among cardiovascular diseases, atrial fibrillation is a common cause of ischemic strokes and pose clinical challenges in patients with hemorrhagic brain tumors, as the primary prevention is systemic anticoagulation. This also complicates treatment for patients with an inherited coagulopathy.

Tumor-associated risk factors for stroke include the tumor size, location, and the biology of the neoplasm. The association of tumor volume relates to the risk of direct or edema-related compression of blood vessels resulting in either diminished blood flow or complete occlusion of arteries. These effects will compromise the downstream blood supply resulting in hypoperfusion or ischemic stroke. In addition, malignant cancers can also invade vessels directly which may lead to intracranial hemorrhage or tumor induced occlusion [6]. Certain brain cancers produce coagulation promoting factors that are associated with systemic or local thrombosis. For example, glioblastoma promotes a prothrombotic environment by the secretion of tissue factor and podoplanin often manifest as intravascular thrombosis within the tumor tissue and by deep venous thrombosis in the peripheral circulation [7, 8].

At some point in the disease trajectory, most commonly at diagnosis, patients undergo a neurosurgical procedure that involves a craniotomy with tumor resection. Stroke is a well-known postoperative complication, particularly in patients with underlying risk factors. However, even patients without stroke risk factors can have a vascular complication, so all surgical consent discussions must include the risk of stroke. These vascular events frequently occur in the postoperative period in brain cancer patients as it was reported by an observational study of 3889 glioma patients by the German Glioma Network. They revealed that a total of 193 patients had a cerebrovascular event during the observational period (between October 2004 and January 2010). Among the 70 patients with an ischemic stroke, 61% presented within 30 days of surgery. Among the 123 patients with an intracranial hemorrhage, 68% happened within 7 days after tumor resection [9]. Patients with postoperative ischemic stroke were younger and had fewer vascular risk factors than patients who suffered an ischemic stroke outside of the postoperative period. Epidural hematomas developed more frequently in the postoperative period, but subdural and subarachnoid hemorrhage were seen more commonly in the non-perioperative period. Severe disability (modified ranking scale above 3) was registered more often in patients with postoperative bleeding (49%) compared to patients who had an intracranial hemorrhage in the non-postoperative period (21%). Additionally, intraoperative hemodynamic instability may also play a role in the development of stroke in the perioperative period.

Cervicocranial radiotherapy accelerates atherosclerotic disease in the vessels involved in the radiation field and leads to radiation vasculopathy, which is a strong risk factor for cerebrovascular complications. Cancer survivors who receive radiation treatment in childhood may acquire additional vascular risk factors in their lifetime that increase the chances of stroke, as well as being at risk for a progressive vascular disease process due to radiation called moyamoya. Adults with existing vascular injury (i.e., atherosclerosis) at the time of radiation treatment have an even higher risk for severe vascular complications. In addition, radiation injury to cerebral vasculature may rarely lead to delayed aneurysm formation in the radiation field. These aneurysms usually have abnormally weak walls that make them very susceptible for rupture [10]. As the development of radiation-induced vasculopathy is time-related and typically a late complication, patients with benign brain tumors or low-grade malignancies who are most likely to have prolonged survival are also most likely to develop stroke as it typically develops several years after their cancer treatment [11]. Conventional radiation is delivered using photons; therefore, most of our knowledge about radiation-induced vasculopathy is in the setting of photon therapy. Proton radiation, a relatively new option, lacks long-term safety data regarding vascular toxicity and injury. Currently, radiation treatment remains the cornerstone of therapy for most brain tumors, but consideration of late effects such as vascular injury should be considered particularly in patients with lower grade tumors where there is potential for long-term survival.

Chemotherapeutic agents may lead to stroke by multiple mechanisms, including direct vessel injury or hematologic changes. For example, temozolomide, the most used chemotherapy in neuro-oncology, is known to lead to thrombocytopenia (grades 3 and 4 in 3–11% of the patients) and therefore increases the risk for (intracranial) hemorrhage [12]. Cisplatin, used for the treatment of medulloblastoma and other primary brain tumors, has been reported to increase the risk for stroke [13]. Intrathecal administration of methotrexate and other agents where there is direct exposure to brain surfaces can lead to stroke-like events that should be considered as a differential diagnosis in appropriate cases [14].

Supportive medications used in the care of brain tumor patients may have properties that increase the risk for stroke. Most importantly, bevacizumab, used in neuro-oncology as a steroid sparing agent or for the treatment of radiation necrosis, has been associated with ischemic stroke and intracranial hemorrhage [15]. Corticosteroids, specifically dexamethasone, often leads to drug induced diabetes mellitus and hypertension, thereby increasing stroke risk by creating or exacerbating hyperglycemia and/or hypertension. Finally, thromboembolic disease including deep venous thrombosis (DVT) and pulmonary embolus (PE) is common in patients with malignant brain tumors. The current standard of care is systemic anticoagulation thereby increasing the risk and severity of an intracranial hemorrhage. For this reason, prophylactic anticoagulation is not recommended for patients with primary brain cancers even though the incidence of DVT and PE is greater than 30%.

### Presentation and Differential Diagnosis

Strokes can be classified into two main categories: ischemic strokes (due to occlusion of an artery or arteries that supply the brain tissue) and hemorrhagic strokes (due to bleeding from a cerebral blood vessel). The clinical presentation of stroke depends on the region of the brain that is affected by the loss of blood perfusion in the specific vascular territory. Although called “asymptomatic stroke” and the patient reports no symptoms, functional deficits may be appreciated by thorough clinical evaluation. Because of the subtle clinical signs (for example, decline in complex cognitive functions), these so-called asymptomatic strokes are often found on surveillance MRI in brain cancer patients. In this patient population, this finding may be underreported and less likely to be clinically addressed. However, an asymptomatic stroke may portend an underlying vascular pathology that may lead to a devastating vascular event and therefore requires evaluation and treatment to reduce the risk of a recurrent and potentially catastrophic stroke. Symptomatic strokes, in general, present with the sudden onset of focal neurologic deficits. High blood pressure is frequently seen with acute stroke. In contrast to ischemic stroke, the most common initial symptoms at the onset of intracranial hemorrhage (hemorrhagic stroke) are headache, vomiting, and seizures. Clinical signs may also include change in mental status, hemiparesis, and signs of increased intracranial pressure. In comparison, with ischemic strokes, seizures, significant change in mental status, and vomiting are less frequent.

In addition to the well-known stroke risk factors, multiple unique mechanisms can lead to ischemic strokes in patients with brain cancer and the signs of a stroke may mimic other frequent neurologic events in patients with brain tumors including seizures. As a result, recognizing signs of ischemic stroke may be challenging in this patient population. Kamiya-Matsuoka reviewed 60 cases of ischemic strokes in patients with glioma [16]. They reported that 17% of the patients had a stroke pre-operatively, 53% suffered stroke within 2 weeks of surgery, and 33% had a stroke more than 2 weeks after surgery. Also, they found that most of the ischemic strokes localized adjacent to the resection cavity in patients who underwent a surgical procedure (either resection or biopsy). Furthermore, the onset of a new focal neurologic deficit has an extensive differential diagnosis in a brain tumor patient besides stroke, including tumor progression, brain edema, seizures, and SMART (stroke-like migraine attacks after radiation therapy) syndrome. SMART syndrome is both intriguing and clinically challenging. It is a rare and delayed complication of radiotherapy that often presents with headaches, seizures, and cortical signs (such as aphasia, hemiplegia, hemianopia) related to the area of prior radiation [17]. In the acute phase, imaging may be normal but cortical enhancement with mild mass effect can be well visualized on MRI few days after symptom onset. These imaging findings eventually resolve, often requiring high dose corticosteroids and anticonvulsant management. Patients typically have improvement of symptoms if not full recovery over several weeks or months. The episode may recur later without any predictable pattern.

### Diagnostic Considerations

**Fig. 1:**
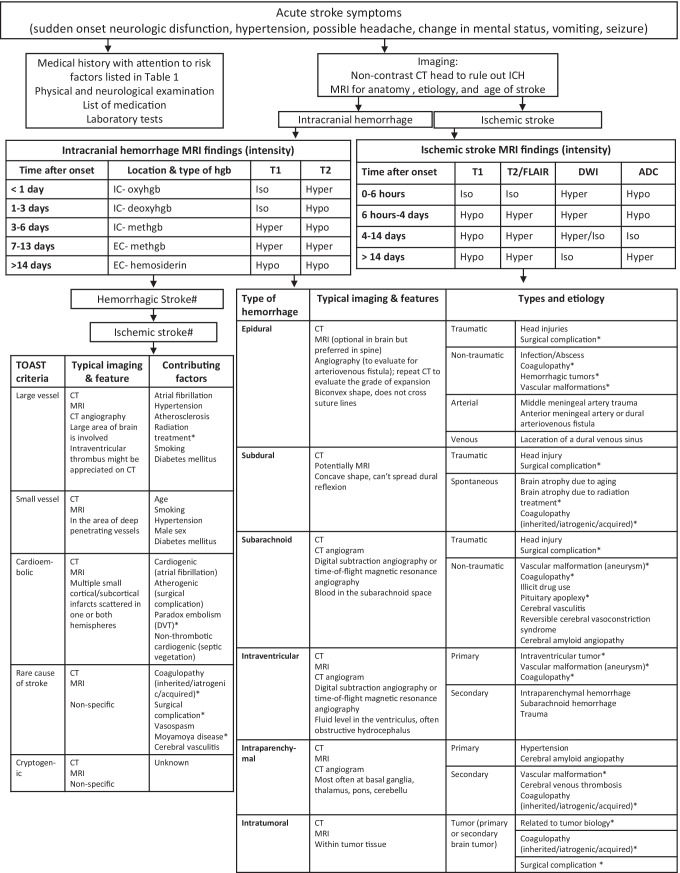
Flowchart of the management of stroke in primary brain cancer patient ADC: apparent diffusion coefficient; DWI: diffusion weighted imaging; EC: extracellular; hgb: hemoglobin; IC: intracellular; *increased risk in patients with brain cancer

An acute change in neurological status or function irrespective of an existing cancer diagnosis requires immediate attention and further investigation (Fig. 1). A careful neurologic examination will help to provide neuroanatomic localization and the history of the event will help to further refine the differential diagnosis. Imaging of the appropriate region of the central nervous system is essential and acute changes in the limbs or torso should bring spinal cord processes into the diagnostic possibilities, underscoring the importance of the neurologic examination. While magnetic resonance imaging provides better anatomic and etiologic information, it is more time consuming. Conversely, in the setting of a rapid neurologic deterioration, a non-contrast CT of the head is the fastest and most easily available imaging modality to rule out acute intracranial hemorrhage. Despite the limited resolution, CT can distinguish multiple types of hemorrhage including epidural, subdural, subarachnoid, intraparenchymal, intratumoral, and intraventricular hemorrhage. The location of the blood can also provide information about the potential origin of bleeding. Epidural hemorrhages are more frequently due to injury of the middle meningeal artery after a trauma, yet venous bleeding from a skull fracture can also occur especially in the pediatric population. Subdural hematomas originate from a tear the bridging veins and most frequently result from head trauma. However, particularly in the setting of brain atrophy often found in elderly as a late consequence of cranial radiation, subdural hematomas can occur spontaneously. Subarachnoid hemorrhage from a defect in an intracranial artery can be traumatic or non-traumatic as well as aneurysmal versus non-aneurysmal. In patients with brain tumors, accidental injury to an artery during tumor resection or an aneurysm associated with infectious endocarditis are distinct causes for subarachnoid hemorrhage among brain tumor and all cancer patients, respectively. Specialized imaging studies such as CT angiography are required to help determine the location and cause of the hemorrhage. Importantly, intraventricular hemorrhage, which is most commonly seen with aneurysmal subarachnoid hemorrhage or hypertension-related intracerebral hemorrhage may cause obstructive hydrocephalus and usually results in poor outcome. Intraparenchymal hemorrhage can have multiple contributing and causative factors including hypertension, arterial venous malformation, aneurysm, amyloid angiopathy [iatrogenic], and coagulopathy. This is the most commonly seen type of hemorrhagic stroke. Intratumoral hemorrhage is a type of intraparenchymal bleeding which is most seen in pituitary adenomas and in high-grade tumors, including glioblastoma as well as some brain metastases such as melanoma, choriocarcinoma, and renal cell cancer. Although certain cancer types are more prone to bleeding, additional factors like thrombocytopenia, coagulopathy, certain medications may also contribute to the risk of intratumoral bleeding.

The diagnostic evaluation of intracranial bleeding includes testing for an underlying coagulopathy, either iatrogenic (anticoagulant medication, thrombocytopenia), or hereditary (i.e., Factor V Leiden syndrome). Importantly, MRI can be very useful in determining both the anatomic localization as well as determine the age of the hemorrhage (Fig. 1). Blood undergoes a specific transformation (oxyhemoglobin- > de-oxyhemoglobin- > intracellular methemoglobin- > extracellular methemoglobin- > hemosiderin) over a very well-established timeline and the age determined by well described MRI changes seen on T1 and T2 sequences. MRI-GRE hyperintensity, denoting iron-rich hemosiderin, is a sensitive but non-specific sign of intracranial bleeding.

Although rapid and readily available, non-contrast CT head is less helpful in the detection of ischemic strokes. Hypodensity, on a non-contrast CT head image, may represent an acute ischemic stroke, especially with corresponding clinical history and signs, although tumor edema may result in similar imaging findings. Again, MR imaging is superior as it provides information about the location and about the age of ischemic stroke (Fig. 1). In the hyperacute phase (up to 6 h after stroke onset), diffusion-weighted imaging (DWI) is bright while the corresponding apparent diffusion coefficient (ADC) is dark. T1 and T2 sequences are isointense. In the acute phase, T1 turns permanently dark and T2 sequences become permanently bright while DWI and ADC remain bright white and dark, respectively. After 4 days of stroke onset, DWI is isointense or bright while ADC is isointense. In the chronic phase (> 14 days), DWI becomes isointense while ADC turns bright. Although these guidelines for stroke evolution are helpful, in the setting of a pre-existing brain tumor, it can be challenging to distinguish tumor tissue from ischemic stroke on MR images. While time often clarifies the etiology of the abnormality, CT angiography or MRI perfusion studies may provide additional information in the acute setting. These studies help distinguish tumor from stroke because cancer cells have increased metabolic activity and require increased blood supply. Conversely, ischemic stroke correlates with decreased blood flow and volume in addition to disrupting the white and gray matter differentiation.

### Acute Treatment

The development of a stroke, whether ischemic or hemorrhagic, requires immediate attention and medical management [18]. However, there are a paucity of treatment recommendations for managing acute stroke in cancer patients. Similar to the evaluation of other patients with stroke, it is important to collect accurate medical history (including chronic diseases and comorbidities, recent surgical procedures and past treatments, and social history with attention to illicit drug use) and a complete list of medications (including anticoagulation, antiplatelet therapy, and chemotherapy). Once the patient is deemed to be medically stable, imaging is required. As described above, rapid acquisition of a non-contrast head CT can quickly determine if there is an intracranial hemorrhage which may require an emergent neurosurgical intervention. MR imaging should also be considered as it provides better anatomic information as well as assessment of the event timeline.

The finding of a hemorrhage, particularly subarachnoid, warrants a vascular imaging study (CT or MR angiography). Medical management should also be implemented quickly as most patients with intracranial hemorrhage present with elevated blood pressure which should be treated with the goal of reaching systolic blood pressure of 140 mm Hg (within 1 h if systolic blood pressure is < 220 mm Hg). Additional testing includes a complete blood count, coagulation studies, as well as other routine laboratory studies to determine if there are other contributing factors such as hepatic or renal failure or hyperglycemia. For hemorrhagic strokes, thrombocytopenia (typically chemotherapy-induced) or excessive anticoagulation (often seen with warfarin) will be uncovered by the CBC and determination of the INR, respectively. Thrombocytopenia in setting of intracranial hemorrhage warrants consideration of platelet transfusion, using a threshold that is higher than standard because of the risk of ongoing hemorrhage. Similarly, for patients on warfarin or other vitamin K antagonists, rapid correction of elevated INR is recommended by using fresh frozen plasma, vitamin K, prothrombin complex concentrates, activated PCC FEIBA, or recombinant activated factor VIIa. Hemostatic correction of direct oral anticoagulants may require the use of prothrombin complex concentrates, activated PCC FEIB, idarucizumab, or coagulation factor Xa (recombinant), inactivated-zhzo. Despite the presence of factors contributing to blood clotting issues, these patients remain at high risk of DVT; therefore, intermittent pneumatic compression should be used as prophylaxis. Blood glucose should be kept within normal ranges because both hypoglycemia and hyperglycemia lead to worse outcome. Resuming anticoagulation after intracranial hemorrhage is questionable, especially in the malignant glioma population where tumor-related coagulopathy and the risk of intracranial re-bleeding need to be carefully balanced [19].

In the case of ischemic stroke that is confirmed by non-contrast CT head and brain MRI, a standard stroke work-up should be done preferably within 48 h of symptoms onset. This includes, EKG, blood work (including complete blood count, lipid panel, glucose level, HgbA1c, prothrombin time, partial thromboplastin time), and carotid imaging in selected cases. As above, blood pressure control and blood glucose management are important along with DVT prophylaxis. MR or CT angiography may help to determine the vascular pathology that led to the stroke. Echocardiogram may be considered if cardiac etiology is suspected, particularly if the areas of brain ischemia are most consistent with an embolic etiology.

Direct treatment of an acute ischemic stroke is more complicated in patients with cancer, particularly those with intracranial tumors. Whereas tPA is the standard of care treatment for eligible acute stroke cases with onset within 3 h or in 4.5 h in selected patients, those with intracranial tumors are excluded from consideration because of the concerns for hemorrhage. However, there is little information on the outcome of stroke thrombolysis in patients with brain cancer as the literature mostly contains case reports and a larger dataset from the Nationwide Inpatient Sample of the Healthcare Cost and Utilization Project [20–22]. Available data suggest that thrombolysis may be safe for patient with benign brain tumors with similar rate of complications as in the general population; conversely, use of tPA might lead to increased mortality in patients with malignant brain cancer.

### Prevention

As with all patients at risk for stroke, preventive measures are important to decrease the number of strokes in brain cancer patients and improve their quality of life as well as life expectancy. Table 2 summarizes these preventive measures.

**Table 2:** Stroke prevention in patients with primary brain cancer

Risk factor	Primary prevention	Secondary prevention
Hypertension	Regular screening Blood press control as needed Education	Regular screening Goal of < 130/80 Education
Diabetes mellitus	Regular screening Blood glucose control as needed Education	Regular screening Goal of HbA1c < or =7% Diabetes self-management Education
Hyperlipidemia	Regular screening Medical management as needed	Regular screening LDL-C of < 70 mg/dL
Obesity/abdominal fat	Regular screening Education Mediterranean-type diet Physical activities	Regular screening Medical nutritional therapy Behavioral lifestyle-modification program Mediterranean-type diet
Smoking history	Smoking cessation Education	Smoking cessation Education
Lack of physical activity	Education Support	Physical therapy as needed Education Support
Cardiovascular diseases	Screening	Screening as needed and medical management
Genetic risk factors	Screening as needed	Screening as needed
Local vessel compression	Neurosurgical evaluation	Neurosurgical evaluation
Vessel erosion by cancer	Neurosurgical evaluation	Neurosurgical evaluation
Autocrine factors leading to coagulability	Education about signs of deep venous thrombosis and pulmonary emboli	Medical treatment
Vessel injury during brain operation	Select high volume centers	Rehabilitation
Anesthesia related cardiovascular changes	Select high volume centers Pre-operative screening	Rehabilitation
Radiation-induced microvascular changes in small vessels	Radiation planning	Medical management
Radiation-induced microvascular changes in large vessels	Radiation planning	Medical management
Aneurysm formation	Screening Neurosurgical evaluation	Neurosurgical evaluation Rehabilitation
Temozolomide	Education regarding potential side effects, regular blood work	Medical management
Cisplatin	Education regarding potential side effects	Medical management Rehabilitation
Bevacizumab-related coagulopathy	Education regarding potential side effects	Education regarding potential side effects
Corticosteroid	Regular blood glucose and blood pressure check and treatment as needed	Regular blood glucose and blood pressure check and treatment as needed
Antithrombotic agents	Education regarding potential side effects	Education regarding potential side effects

Given the increased risk of vascular events in patients with brain tumors, targeted screening and medical management should start as early as the diagnosis of brain cancer and continue through the patient’s lifetime. Many of the common stroke risk factors can be medically managed or modified by lifestyle changes. Although difficult to quantify, the risk of a vascular complication from the tumor resection is likely lower with a specially trained neurosurgeon at a center of excellence. Decisions regarding radiation therapy and chemotherapy, particularly those patients with many stroke risk factors should be made after assessing the risks and benefits of the cancer treatment. Additionally, patients and families should be educated regarding the potential side effects of medications so they can recognize early signs and get medical attention as soon as possible.

Patients with primary brain tumors often develop strokes from causes that are not typical in the general population. Incorporating secondary preventive measures based on the identification and management of the stroke etiology may maximize the potential for recovery and minimize the recurrence of stroke which is high in this patient population [23]. These efforts should be carried out in an organized way, often including multiple specialists and health care providers [24].

## Conclusion

The death rate from stroke is one of the great success stories in medicine as it has decreased in the USA by 77% between 1969 and 2013 [25], as a result of trans-disciplinary efforts and therapeutic advances. For example, evidence-based guidelines provide clear directions on how to control stroke risk factors. Primary care providers along with medical subspecialists play a significant role in the implementation and maintenance of these recommendations. Also, the public has greater awareness of stroke risk factors and clinical signs of an acute stroke because of nationwide educational campaigns. Moreover, FDA approval of tPA in 1995 and the continuous advances in endovascular techniques contribute to improved stroke outcomes.

The roadmap to reduce death from stroke in the general population could be used as a template to improve outcomes of stroke in brain cancer patients as well as other patients with cancer. First, the identification and management of stroke risk factors specific to this patient population could improve outcomes. This requires collective efforts and cooperation among many specialists and health care providers who are involved in the care of patients with brain tumors. In addition to managing the classic stroke risk factors as early as the diagnosis of brain tumor, cancer treatment–related stroke risk factors should be also clinically addressed. Although many contributing factors have been identified, the exact pathogenic mechanisms of these processes and their treatment options are still not fully understood. Research focusing on the identification of specific mechanisms of treatment-induced vascular changes and on the potential discovery of biomarkers could allow early identification and treatment of patients who are at increased risk for stroke due to their brain cancer treatment. Increased awareness of the potential side effects (including long-term neurologic complications) of different treatments that are considered for a patient should be an important part of the discussion of treatment options. This is especially important for potential long-term survivors who have higher risk of developing vascular changes many years after treatment. Finally, the acute treatment of stroke in brain cancer patients is an area of unmet need since current stroke guidelines exclude patients with all intracranial tumors from receiving thrombolysis treatment. Grouping all brain tumor types as an exclusion criteria for study inclusion in the landmark tPA trial, whether a low-grade or benign tumor or a high-grade aggressive malignant tumor, may have resulted in lost opportunities to institute effective and perhaps a lifesaving therapeutic option that perhaps should only be contraindicated in patients with particularly types or grade of tumor. In this context, the only available, but limited literature suggests that patients with low-grade brain tumors have no increased risk of complications after thrombolysis compared to the general population. However, current published guidelines do not make this distinction, underscoring the need for additional studies that help refine guidelines, thereby optimizing treatment for these patients with uncommon, but heterogeneous tumors. This additional information combined with a systematic approach to diagnosis, risk management, and treatment as we have outlined may help to improve patient outcomes, emulating the great success in stroke management.
